# Solvent-Induced Lag Phase during the Formation of Lysozyme Amyloid Fibrils Triggered by Sodium Dodecyl Sulfate: Biophysical Experimental and In Silico Study of Solvent Effects

**DOI:** 10.3390/molecules28196891

**Published:** 2023-09-30

**Authors:** Gabriel Zazeri, Ana Paula Ribeiro Povinelli, Nathália Mariana Pavan, Alan M. Jones, Valdecir Farias Ximenes

**Affiliations:** 1 Federal Institute of Education, Science and Technology of Mato Grosso (IFMT), Campo Novo do Parecis 78360-000, Brazil; anapovinelli@outlook.com; 2 Department of Chemistry, Faculty of Sciences, São Paulo State University (UNESP), Bauru 17033-360, Brazil; nathalia.pavan@unesp.br; 3 School of Pharmacy, Institute of Clinical Sciences, College of Medical and Dental Sciences, University of Birmingham, Birmingham B15 2TT, UK

**Keywords:** lysozyme, amyloid fibril, solvent effect, molecular biophysics

## Abstract

Amyloid aggregates arise from either the partial or complete loss of the native protein structure or the inability of proteins to attain their native conformation. These aggregates have been linked to several diseases, including Alzheimer’s, Parkinson’s, and lysozyme amyloidosis. A comprehensive dataset was recently reported, demonstrating the critical role of the protein’s surrounding environment in amyloid formation. In this study, we investigated the formation of lysozyme amyloid fibrils induced by sodium dodecyl sulfate (SDS) and the effect of solvents in the medium. Experimental data obtained through fluorescence spectroscopy revealed a notable lag phase in amyloid formation when acetone solution was present. This finding suggested that the presence of acetone in the reaction medium created an unfavorable microenvironment for amyloid fibril formation and impeded the organization of the denatured protein into the fibril form. The in silico data provided insights into the molecular mechanism of the interaction between acetone molecules and the lysozyme protofibril, once acetone presented the best experimental results. It was observed that the lysozyme protofibril became highly unstable in the presence of acetone, leading to the complete loss of its β-sheet conformation and resulting in an open structure. Furthermore, the solvation layer of the protofibril in acetone solution was significantly reduced compared to that in other solvents, resulting in fewer hydrogen bonds. Consequently, the presence of acetone facilitated the exposure of the hydrophobic portion of the protofibril, precluding the amyloid fibril formation. In summary, our study underscores the pivotal role the surrounding environment plays in influencing amyloid formation.

## 1. Introduction

Amyloid aggregates result from either protein native structure partial loss or the inability of proteins to reach their native conformation [1]. There are several diseases associated with amyloid aggregates, such as Alzheimer’s [2], Parkinson’s [3], lysozyme amyloidosis [4], and others [3]. These diseases are caused by the total or partial unfolding of a specific amino acid chain, such as the peptide Aβ, α-synuclein protein, or mutant lysozyme protein [5,6]. The partial loss of the native conformation and consequent formation of the aggregate is a process in which the protein cannot reach its organized level, exposing the hydrophobic groups to the solution and inducing the aggregation of these proteins to attain a conformation with lower energy that is consequently thermodynamically more favorable [7]. Although amyloids originate from different proteins, they share a common characteristic, being predominantly composed of beta sheets organized in a “cross-β” structure with the backbone hydrogen bonding parallel to the fibril axis [5,8,9].

In 2022, a review published in *Nature Reviews Neuroscience* [3] brought attention to a crucial question: Why do fibrils with similar morphologies in electron-microscopic images become associated with different neurodegenerative diseases, especially when the same protein forms the amyloid fibrils? The review presented a comprehensive collection of data showing that the environment in which the protein is inserted plays a pivotal role in amyloid formation. Different environmental properties can shift the process toward oligomer or polymorphic amyloid fibril formation. It is now well established that the composition of the environment, including factors such as ions, protein–protein interactions, solvents, temperature, and *p*H, significantly influences the kinetics of protein aggregation [3]. However, the role of solvent hydrophobicity in this process is still not fully understood. Considering that, regardless of the proteins involved, amyloid aggregates exhibit a typical organizational structure (β-sheets), there is an economic aspect to using low-cost proteins in the study of aggregate formation, known as amyloid-like systems [1]. In this work, the lysozyme protein was chosen as an amyloid-like system.

Lysozyme was identified by Alexander Fleming around 1922 when he was analyzing the nasal mucus of a patient suffering from the common cold [10]. Lysozyme can catalyze the hydrolysis of the β-1,4 glycosidic bond found between N-acetylmuramic acid and N-acetylglucosamine in peptidoglycan, a carbohydrate present in bacterial cell walls [5]. This protein can be found in body fluids (tears, saliva, blood serum) and in egg whites. The 3D structures of human lysozyme (PDB 1REX) and hen egg-white lysozyme (HEWL) (PDB 1LYS) exhibit a very high degree of superimposition (see Figure S1). HEWL is a single polypeptide chain (14.3 kDa) comprising 129 amino acid residues with 4 intramolecular disulfide bridges and an isoelectric point near 11.2, making it highly soluble in aqueous media. Human lysozyme consists of 130 amino acids (14.7 kDa) and shares 60% sequence similarity with HEWL [5]. Mutations in the human lysozyme are associated with lysozyme amyloidosis, leading to the deposition of amyloid fibrils in various organs, such as the kidneys, gastrointestinal tract, lymph nodes, blood vessels, spleen, and liver [11]. Consequently, extensive in vitro investigations have focused on unraveling the molecular mechanisms that lead to the aggregation of both HEWL and human lysozyme, resulting in the identification of various conditions that yield amyloid fibrils through distinct pathways.

The use of the surfactant sodium dodecyl sulfate (SDS) as a chemical agent to induce the production of amyloid fibrils has been demonstrated using different proteins [12,13,14]. A requisite of the protocol is the conduction of the reaction at least two pH units below the isoelectric point of the protein. A mechanism based on the electrostatic interaction of the positively charged protein and the negative SDS was proposed [14,15]. To expand the knowledge of this process, we studied, herein, the effect of organic solvents on the SDS-induced aggregation of lysozyme. Molecular dynamics was applied to model the experimental findings.

## 2. Results and Discussion

### 2.1. Experimental Studies

**Figure 1:**
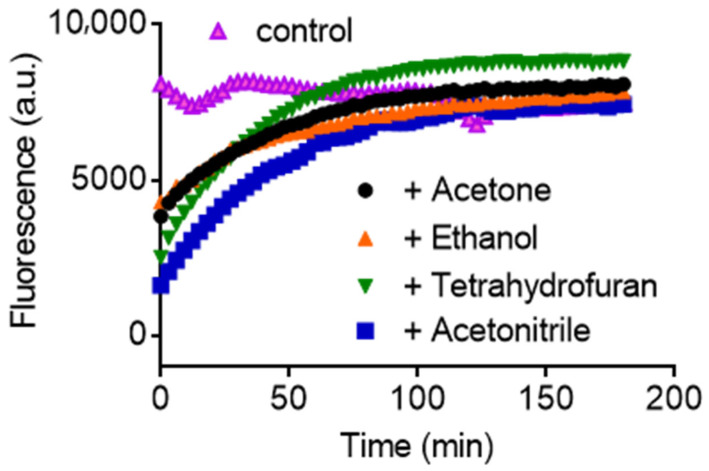
Effect of organic solvents on the SDS-induced aggregation of lysozyme monitored by ThT fluorescence. The control (without solvent, in purple) shows the instantaneous formation of the amyloid fibrils. Reaction conditions: lysozyme (10 µM), ThT (10 µM), SDS (250 µM) in 50 mM phosphate buffer pH 7.0, and solvents (10% *v*/*v*).

**Figure 2:**
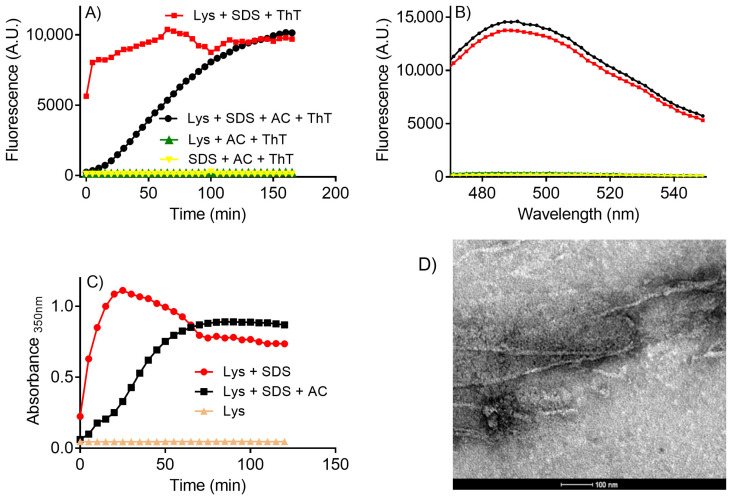
SDS-induced aggregation of lysozyme monitored by ThT fluorescence and turbidity: effect of acetone and controls. (**A**) Reaction kinetics monitored by fluorescence and (**B**) fluorescence spectra. Reaction conditions: lysozyme (10 µM), ThT (10 µM), SDS (250 µM) in 50 mM phosphate buffer, pH 7.0. The reactions were conducted in the absence and presence of acetone (AC, 10% *v*/*v*). (**C**) Reaction kinetics monitored by turbidity. The reaction conditions were the same except for the absence of ThT. (**D**) TEM micrography of lysozyme amyloid fibrils produced by SDS (250 µM) in the presence of acetone (10%). The image was viewed at 12,000x magnification.

Aiming to improve the efficiency of the formation of amyloid fibrils, we studied the combination of SDS and organic solvents (acetone, acetonitrile, ethanol, and tetrahydrofuran THF) with 100% *w*/*w* solubility in water (Table S1) The experiments were conducted in an aqueous buffered solution in the presence or absence of an increasing percentage of organic solvent. Figure 1 shows an unexpected effect in an average result (triplicate); the solvents slowed the rate instead of increasing the relative produced amount. In these experiments, the time-dependent formation of amyloid-like fibrils was monitored based on ThT’s fluorescence. The reactions were triggered by adding SDS. Lysozyme is a cationic protein (*p*I = 11.2), and in the early studies reported in the literature, the reactions were conducted at two pH units below the protein’s pI at pH 9.2 [12,13,14]. The medium’s pH is essential because the proposed mechanism is based on the interaction of the positively charged protein with the negative SDS [13,14,15,16]. Here, we performed the experiments at a neutral pH, which is also effective in generating lysozyme amyloid fibrils [15]. As shown, the total fluorescence intensity, a relative measurement of the amount of amyloid fibrils, was similar in all studied cases. As protein aggregates are poorly soluble [15], the reactions were also monitored by medium turbidity. The experiments were conducted in microplates, and the medium’s turbidity could be observed and caused some heterogeneity. It explains the fluctuations in the fluorescent intensity observed in the control (Figure 1 and Figure 2A). The heterogeneity was decreased by adding solvents in the reaction medium, and a smooth fluorescence increase was noted. Besides reducing the rate of fibril formation, the presence of solvents caused a homogeneity of the medium. The solvents were added at a final concentration of 10% (*v*/*v*) and were chosen based on their water solubility (Table S1). No significant difference was observed among the studied solvents. Hence, acetone, which could be easily removed (Table S1), was selected for further studies.

It is worth noting that organic solvents could interfere with the measurements, for instance, by increasing the fluorescence quantum yield of ThT. However, this was not the case (Figure 2). Figure 2A shows that the fluorescence of ThT was only observed in the presence of both lysozyme and SDS, confirming the correlation between ThT’s fluorescence and the production of amyloid fibrils. Moreover, the presence of acetone did not increase the relative amount of amyloid fibrils (Figure 2B).

The lag phase caused by acetone was not exclusive to the ThT’s fluorescence assay. Indeed, the same behavior was observed by monitoring the medium turbidity at 350 nm, which was used as an analytical approach to study the formation of protein aggregates [14]. Thus, the solvent’s effect observed here is unrelated to any fluorescent assay bias (Figure 2C). Finally, the formation of amyloid fibrils was confirmed through TEM (Figure 2D).

**Figure 3:**
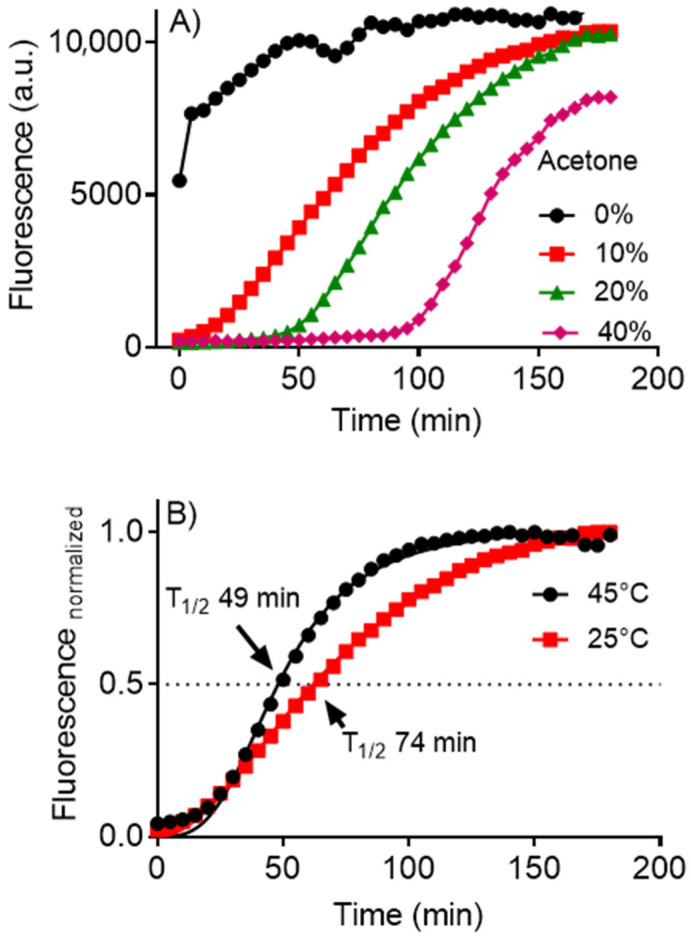
Lag phase induced by acetone concentration on SDS-induced aggregation of lysozyme and effect of temperature: (**A**) Time-dependent profile as a function of acetone concentration. (**B**) Effect of temperature at fixed acetone concentration (10%). Reaction conditions: lysozyme (10 µM), ThT (10 µM), SDS (250 µM) in 50 mM phosphate buffer, pH 7.0.

The significant experimental finding described in this report was the solvent’s concentration-dependent effect in forming amyloid fibrils (Figure 3A). A clear lag phase was observed and correlated with the percentage of acetone used in the assay. This finding suggests that the presence of acetone in the reaction medium impeded the organization of the denatured protein into the fibril form. Thus, as the acetone is removed, the formation of amyloid fibrils follows its natural course, as has been documented [12,13,14,15]. It also suggests that the formation of amyloid fibrils is a favorable pathway for the solvent-denatured protein in the presence of SDS.

To reinforce the proposal that acetone impedes the formation of fibrils, the experiment was performed at a higher temperature, which would favor the evaporation of acetone. Figure 3B compares the T_1/2_ (temperature for half-maximum of fluorescence intensity) to form fibrils at 25 °C and 45 °C. As depicted, the faster formation at 45 °C is consistent with the necessity to eliminate acetone from the medium as a condition for fibril formation.

**Figure 4:**
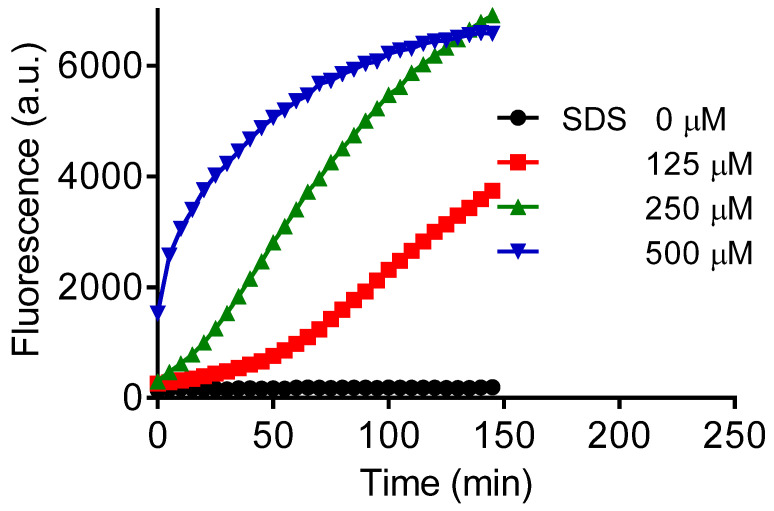
Lag phase decreased by increasing SDS. Reaction conditions: lysozyme (10 µM), ThT (10 µM), acetone 10%, SDS (0–500 µM) in 50 mM phosphate buffer, pH 7.0.

The role of SDS as an inductor of amyloid fibril formation was reinforced by measuring its effect in the presence of acetone (Figure 4). The concentration of SDS was increased, and the solvent concentration was kept constant. Corroborant with the expectation, the lag phase was decreased by increasing SDS, which partially circumvented the inhibitory effect of acetone.

### 2.2. In Silico Studies

Based on the experimental results, which revealed certain solvents as delay agents in the aggregation process, an interest arose in uncovering the molecular mechanism behind this effect. To pursue this idea, we focused on investigating the early stages of the amyloid formation process by studying the stability of lysozyme protofibrils in different solvents.

As lysozyme protofibril tertiary structure has not been deposited in the PDB, the first step was to model its structure. This task was accomplished considering that amyloid protofibrils share a typical structure. We performed the modeling using lysozyme native structure (1LYS and 1REX) and the protofibril (PDB 2BEG) (Figures S1 and S2), followed by subjecting it to molecular dynamics simulations (1000 ns) under the same conditions as the experimental solutions.

**Figure 5:**
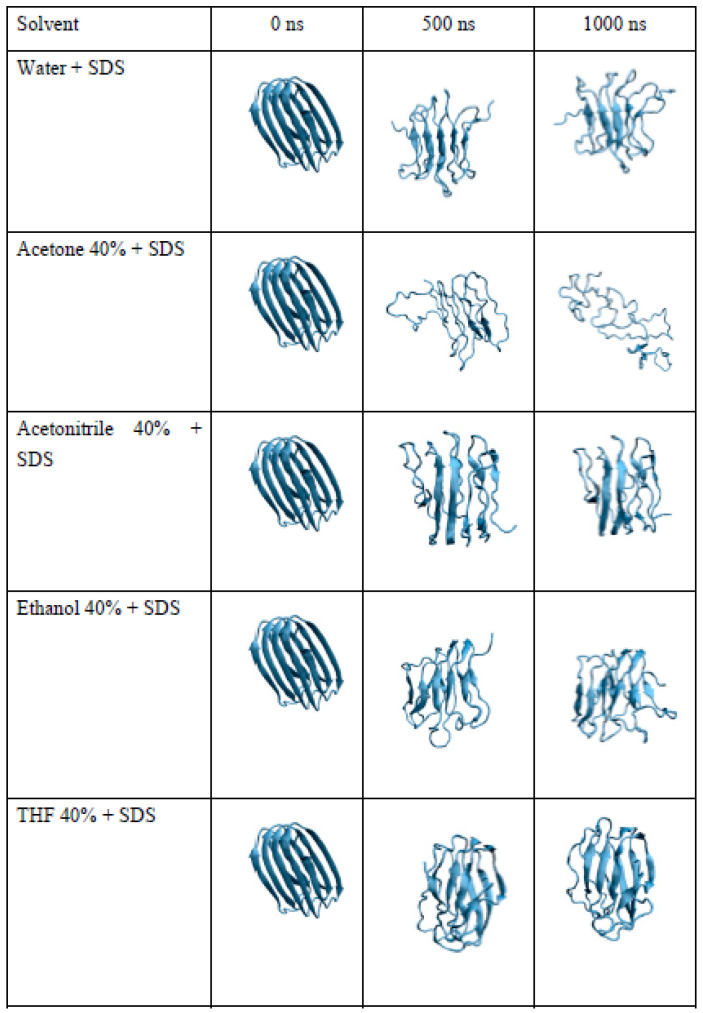
Three-dimensional structure of modeled lysozyme protofibril on different solvents (water, acetone, acetonitrile, ethanol, THF) at three different stages of molecular dynamics simulation (0, 500, and 1000 ns).

Figure 5 shows the 3D protofibrils at 0, 500, and 1000 ns in different solvents. In agreement with the experimental findings, it is notable that acetone caused intense protofibril destabilization, with a complete loss of the β-sheet conformation.

**Figure 6:**
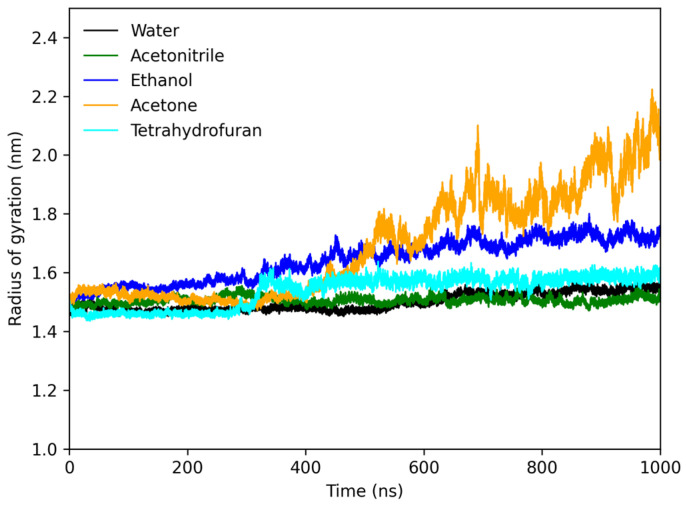
Radius of gyration plot of lysozyme protofibril in water (black), acetonitrile (green), ethanol (blue), acetone (orange), and tetrahydrofuran (cyan).

The protofibril’s gyration (RG) radius was monitored in the sequence. Figure 6 illustrates that, over a 1000 ns simulation period, the protofibril’s RG in water, THF, and acetonitrile exhibited minimal deviation, remaining within a range of values between 1.4 and 1.6 nm throughout the simulation. In contrast, when the protofibril was exposed to ethanol, its RG increased from 1.5 to 1.7 over 500 ns and remained constant until 1000 ns. In acetone, the protofibril’s RG continuously increased during the entire simulation without showing signs of stabilization, unlike in the other solvents. As a result, the protofibril exposed to acetone adopted an open conformation, exposing its entire structure to the solvent. This feature can be observed in Figure 5; the protofibril lost its organized β conformation and transformed into an open, disordered coil. In short, the behavior of the protofibril in acetone indicates that this solvent disrupts the native structure, leading to an open and disordered configuration. In contrast, other solvents provided a more stable and organized conformation throughout the simulation.

**Figure 7:**
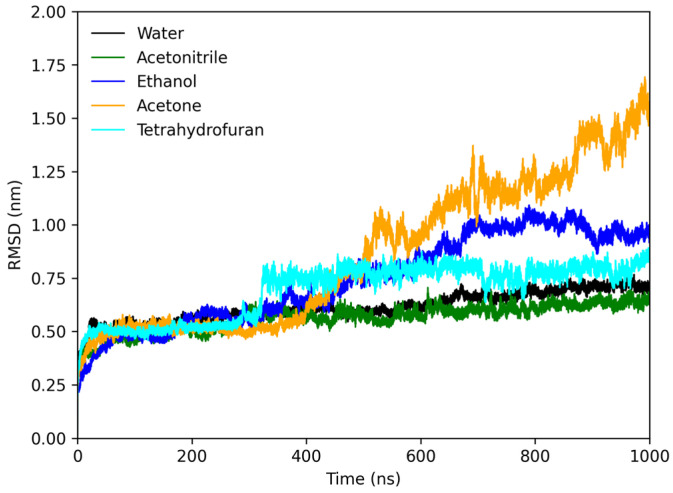
RMSD plot of lysozyme protofibril in water (black), acetonitrile (green), ethanol (blue), acetone (orange), and tetrahydrofuran (cyan).

Figure 7 shows the RMSD plot of the protofibrils in different solvents. According to the results, the protofibrils in water and acetonitrile increased RMSD from 0.25 to 0.50 nm within 50 ns, reaching stability. THF and ethanol had their respective RMSD increased for about 400 ns, reaching stability. In contrast, the protofibril in acetone had the RMSD increase throughout the simulation, showing total destabilization of the structure in this medium. These results are in concordance with the RG results and the experimental results.

**Figure 8:**
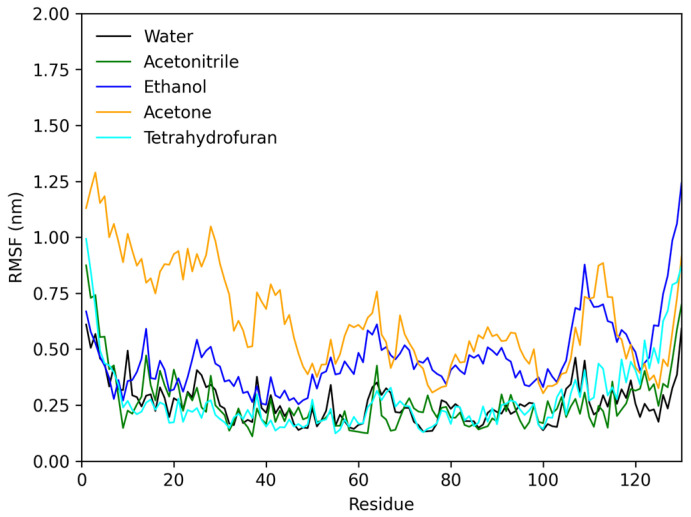
RMSF plot of lysozyme protofibril in water (black), acetonitrile (green), ethanol (blue), acetone (orange), and tetrahydrofuran (cyan).

Figure 8 shows the RMSF of the protofibrils in different solvents; these analyses may reveal the most important amino acid residues in the stabilization of the 3D structure. According to the results, protofibrils in water, THF, and acetonitrile had stable residues without significant deviation on equilibrium position. Comparing the results of protofibrils in ethanol and acetone, both had considerable fluctuation in the position of the residues from 50 to 150. In acetone, only the residues from 1 to 50 had more considerable fluctuation than those in the other solvents. This result suggests that residues from 1 to 50 are the key to molecular structure stability (Figure S3).

**Figure 9:**
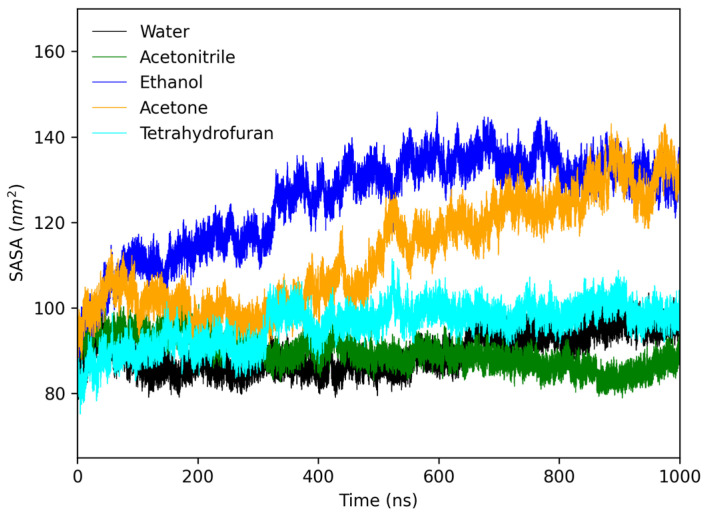
Solvent accessible area (SASA) of protofibril in water, acetone, and THF during 1000 ns of simulation.

The formation of amyloid β structures occurs through the partial loss of their native conformation, leading to the aggregation process in which the protein cannot attain its organized state [17]. Consequently, hydrophobic groups are exposed to the hydrophilic solvent, forcing the proteins to aggregate into a conformation with lower energy, which is thermodynamically more favorable [7]. To investigate this phenomenon, the Surface Area of Solvent Accessible (SASA) was analyzed for the protofibril in the studied media. Figure 9 illustrates that the protofibril in acetone exhibited the highest area accessible to the solvent, suggesting that the hydrophobic portion of the amyloid protofibril found a more favorable environment in acetone compared to other media.

**Figure 10:**
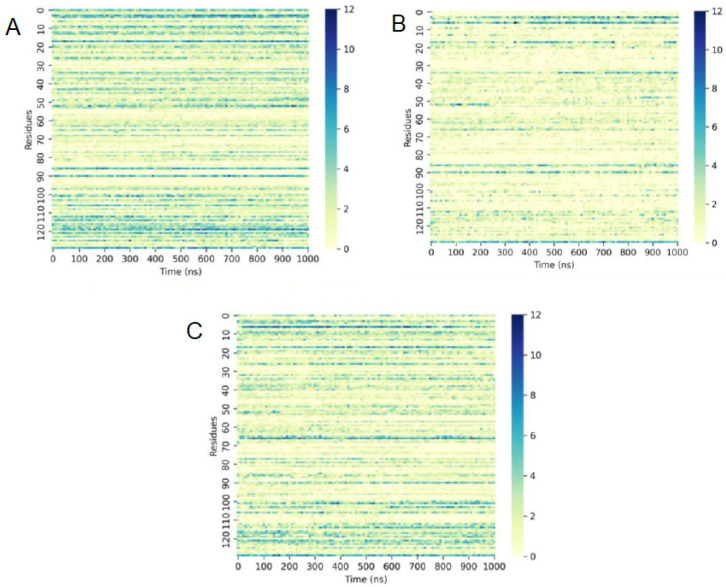
Number of hydrogen bonds performed between water molecules and protofibril residues in (**A**) water, (**B**) acetone, and (**C**) THF during 1000 ns.

**Figure 11:**
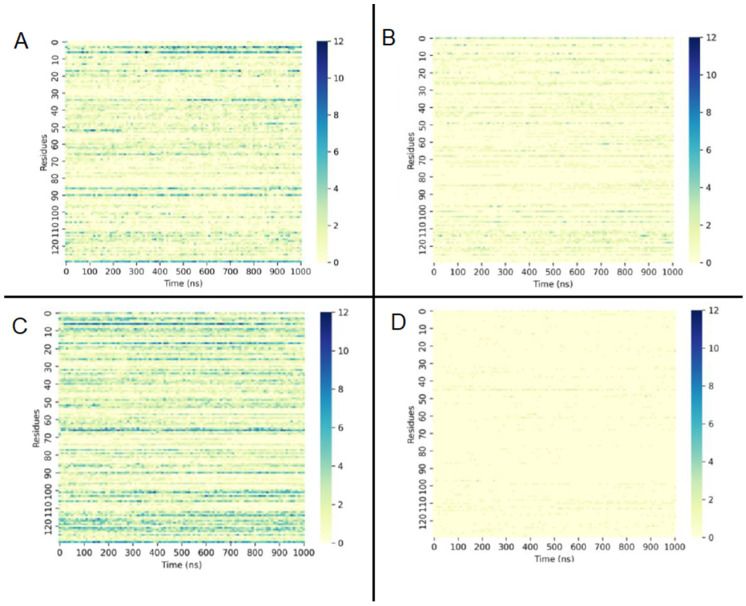
Number of hydrogen bonds formed between (**A**) water molecules and protofibril residues in acetone, (**B**) acetone molecules and protofibril in acetone, (**C**) water molecules and protofibril residues in THF, and (**D**) THF molecules and protofibril residues in THF during 1000 ns.

**Figure 12:**
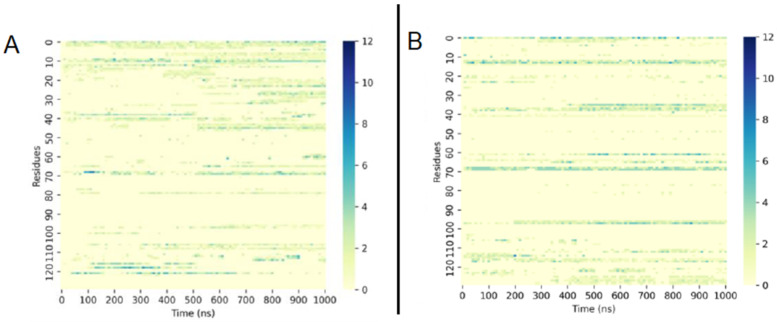
Number of hydrogen bonds formed between SDS molecules and protofibril residues in (**A**) acetone and (**B**) THF during 1000 ns.

Aiming to understand the instability of the protofibril in acetone, the hydrogen bonds between solvent molecules and the protein were examined. Figure 10 reveals that, in water or THF, the number of hydrogen bonds between water molecules and the protofibril’s amino acid residues was much higher than in acetone. Acetone molecules also formed more hydrogen bonds with protofibril residues than THF molecules and fewer hydrogen bonds of water molecules with protofibrils (Figure 11). Moreover, the interaction of SDS molecules with the protein in acetone and THF solution revealed that SDS formed just a few hydrogen bonds with protofibrils in both solutions (Figure 12).

## 3. Materials and Methods

### 3.1. Chemicals and Proteins

Hen egg-white lysozyme (>98%), thioflavin T (ThT), and sodium dodecyl sulfate (SDS) were purchased from Sigma-Aldrich Chemical Co. (St. Louis, MO, USA). Lysozyme was dissolved in 50 mM phosphate buffer at pH 7.0 to give a 1.0 mM stock solution and stored at 4.0 °C. The concentration of the stock solution was determined by its absorbance at 280 nm (43,824 M^−1^·cm^−1^). A 1.0 mM stock solution of ThT was prepared in deionized water. The solvents acetone, ethanol, acetonitrile, and tetrahydrofuran were of spectroscopic grade.

### 3.2. SDS-Induced Formation of Amyloid Fibrils: ThT Fluorescence Measurements

Lysozyme (10.0 μM) was diluted in 50 mM phosphate buffer pH 7.0 at 25 °C with ThT (10.0 µM) in the presence or absence of organic solvents (10, 20, 40%, *v*/*v*). Then, SDS (250 µM) was added to trigger the formation of amyloid fibrils. The reactions were conducted in a 96-well flat-button black microplate (total volume 250 µL), and the ThT fluorescence was measured on a Spectramax M2 microplate reader (Molecular Devices, Sunnyvale, CA, USA) adjusted as follows: excitation at 435 nm and emission at 480 nm. The spectra are an average of three experiments. Figure S4 shows the control of ThT on the solvents, showing that ThT does not fluoresce just in the presence of the solvents.

### 3.3. SDS-Induced Formation of Amyloid Fibrils: Turbidity Measurements

Lysozyme (10.0 μM) was diluted in 50 mM phosphate buffer pH 7.0 at 25° in the presence or absence of acetone (10%, *v*/*v*). Then, SDS (250 µM) was added to trigger the formation of amyloid fibrils. The reactions were conducted in a 96-well flat-button transparent microplate (total volume 250 µL), and light absorption was measured on a Spectramax M2 microplate reader adjusted at 350 nm.

### 3.4. Transmission Electron Microscopy (TEM)

The micrographs were obtained using a transmission electron microscope—FEI TECNAI G^2^ F20 HRTEM operating at 200 kV. The fibril was produced by reacting 10 µM lysozyme with 250 µM SDS in 50 mM phosphate buffer pH 7.0 and acetone 10% (*v*/*v*). After 2.5 h, the sample was 1:1 diluted in phosphate buffer and submitted to microscopy. The positively charged grid was loaded with 3.0 µL of the reaction mixture, and the excess was removed after 30 s. Then, the grid was washed with deionized water (3x) and dried, and uranyl acetate 2% (3 µL) was added and dried for 3 min before analysis.

### 3.5. Computational Studies

The structural modeling for the lysozyme protofibril structure was built using MODELLER [18,19] with sequence–sequence, sequence–profile, and PSI-Blast as fold assignment methods. Peptide Aβ protofibril 1-42 (2BEG) was used as a template for both structures of native lysozyme (1LYZ, 1REX) (Figure S1). Both templates generated the same protofibril structure (Figure S2). The modeled structure was subjected to molecular dynamics simulation with a GROMOS54a7 force field by Gromacs v.5.1.4 [20]. Lysozyme was placed in a rectangular box, solvated with simple point charge water (SPC), and neutralized with NaCl in different compositions: (water, acetone (40%), acetonitrile (40%), ethanol (40%), and tetrahydrofuran (40%)). SDS and water were present in all cases. The molecules were inserted in the box using the gmx insert molecules program. The energy minimization was performed with the steepest descent. The first equilibration step was performed in an NVT ensemble for 100 ps. The system was coupled to the V-rescale thermostat [21] at 298 K. All bonds were constrained with the LINCS algorithm [22], the cutoff for short-range non-bonded interactions was set at 1.4 nm, and long-range electrostatics were calculated using the particle-mesh Ewald (PME) algorithm [23]. The second equilibration step was performed in the NPT ensemble coupled to a Parrinello–Rahman barostat to regulate the pressure for 100 ps [24] isotropically. The analyses of RMSD, RMSF, radius of gyration, SASA, and hydrogen bonds were based on an average of three independent simulations of 1000 ns with the gromacs programs gmx rms, gmx rmsf, gmx gyrate, gmx sasa and gmx hbond, respectively (https://manual.gromacs.org/archive/5.0/programs/byname.html, accessed on 1 august 2023). The molecular dynamics images were generated with visual molecular dynamics (VMD) [25].

## 4. Conclusions

Acetone impeded the SDS-induced aggregation of lysozyme into the amyloid-like structure. The lag phase could be correlated with the percentage of the solvent in the medium. As acetone evaporated, aggregation took place. In agreement, the in silico studies showed that acetone was more effective in destabilizing the protofibril protein model, leading to an open and disordered configuration. The solvation layer of the protofibril in acetone solution is significantly lower than in other media, resulting in fewer hydrogen bonds. Consequently, the acetone medium provides a more favorable environment for the exposure of the hydrophobic portion of the protein. The increased RG, RMSD, and RMSF in acetone corroborated these data. The SASA study followed the same trend, showing that, in acetone, the protofibril model presented the highest area accessible to the solvent, suggesting that the hydrophobic portion of the amyloid protofibril found a more favorable environment in acetone compared to the other studied solvents. In conclusion, the in silico results indicated the molecular mechanism and explained the experimental results in which the presence of acetone in the reaction medium impeded the organization of the denatured protein into the fibril form.

## Supplementary Materials

The following supporting information can be downloaded at https://www.mdpi.com/article/10.3390/molecules28196891/s1: Table S1: Physicochemical characteristics of solvents molecules (Phenomenex table and [26]). Figure S1: Structural alignment of 1LYZ (cyan) and 1REX (blue) at two different points of view. Figure S2: Structural alignment of protofibril of 1LYZ (Cyan) and 1REX (Blue). Figure S3: Residues from 1 to 50 of modeled protofibril lysozyme highlighted.
