# Supplementary material for: Solvent-Induced Lag Phase during the Formation of Lysozyme Amyloid Fibrils Triggered by Sodium Dodecyl Sulfate: Biophysical Experimental and In Silico Study of Solvent Effects
Source: Molecules. 2023 Sep 30;28(19):6891. doi: 10.3390/molecules28196891 (PMC10574774; doi:10.3390/molecules28196891)
Supplement: Supplementary file 1 [file molecules-28-06891-s001.zip › molecules-2622492-supplementary.pdf]

molecules

MDPI

Supplementary material

Solvent-Induced Lag Phase during the Formation of Lysozyme Amyloid Fibrils Triggered by Sodium Dodecyl Sulfate: Biophysical Experimental and In Silico Study of Solvent Effects

Gabriel Zazeri [1,ψ,ξ], Ana Paula Ribeiro Povinelli [1,ξ], Nathália Mariana Pavan [3], Alan M. Jones [2,ψ] and Valdecir Farias Ximenes [3,ψ]

1 Federal Institute of Education, Science and Technology of Mato Grosso IFMT, Mato Grosso, Campo Novo do Parecis 78360-000, Brazil; anapovinelli@outlook.com
2 School of Pharmacy, Institute of Clinical Sciences, College of Medical and Dental Sciences, University of Birmingham, Edgbaston, Birmingham B15 2TT, UK
3 Department of Chemistry, Faculty of Sciences, UNESP—São Paulo State University, Bauru 17033-360, Brazil; nathalia.pavan@unesp.br
ψ Correspondence: gabriel.zazeri@outlook.com (G.Z.); a.m.jones.2@bham.ac.uk (A.M.J.); valdecir.ximenes@unesp.br (V.F.X.)
ξ Current address: Department of Chemistry, Institute of Chemistry, University of Campinas UNICAMP, Campinas 13083-970, Brazil.

Citation: Zazeri, G.; Povinelli, A.P.R.; Pavan, N.M.; Jones, A.M.; Ximenes, V.F. Solvent-Induced Lag Phase during the Formation of Lysozyme Amyloid Fibrils Triggered by Sodium Dodecyl Sulfate: Biophysical Experimental and In Silico Study of Solvent Effects. *Molecules* **2023**, *28*, 6891. https://doi.org/10.3390/molecules28196891

Academic Editors: Filipe Menezes, Grzegorz Popowicz and Carmelo La Rosa

Received: 5 September 2023
Revised: 20 September 2023
Accepted: 28 September 2023
Published: 30 September 2023

Copyright: © 2023 by the authors. Licensee MDPI, Basel, Switzerland. This article is an open access article distributed under the terms and conditions of the Creative Commons Attribution (CC BY) license (https://creativecommons.org/licenses/by/4.0/).

Solvents	LogP	Solubility in water (% w/w)	Boiling point (°C)	Dielectric constant
**Acetone**	–0.23	100	56	20.7
**Ethanol**	–0.24	100	78	24.3
**Acetonitrile**	–0.33	100	82	36.2
**Tetrahydrofuran**	0.49	100	65	6.4

Table S1: Physicochemical characteristics of solvents molecules (Phenomenex table and doi.org/10.1007/s002160000467

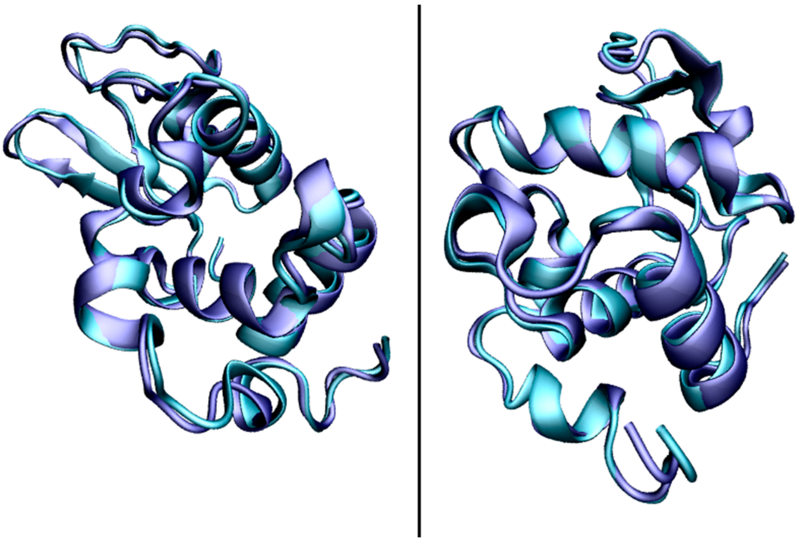

Figure S1: Structural aligment of 1LYZ (Cyan colour) and 1REX (Blue colour) at two different point of views.

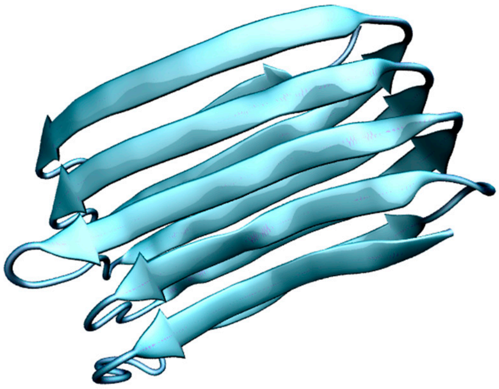

Figure S2: Structural aligment of protofibril of 1LYZ (Cyan colour) and 1REX (Blue colour) .

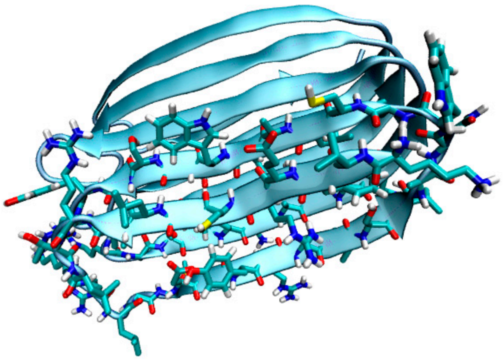

Figure S3: Residues from 1to 50 of modeled protofibril lysozyme highlighted.

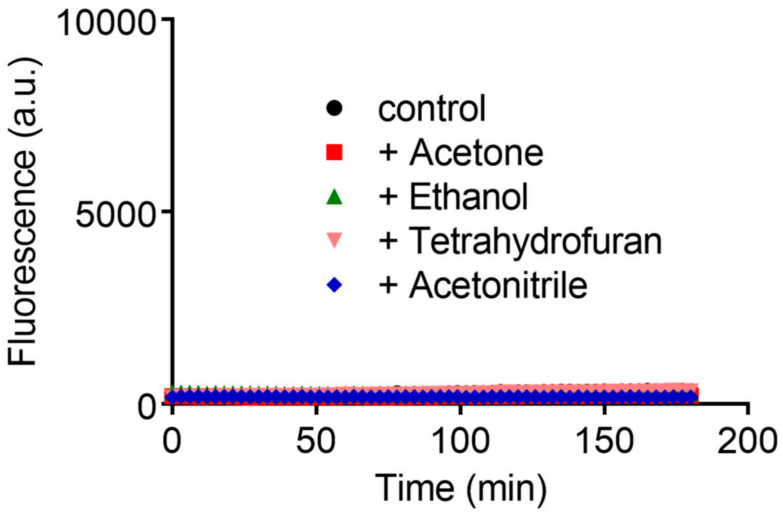

Figure S4: ThT fluorescence in the presence of the different solvents
